# Adaptability, supernaturalness, and the neurocognitive basis of the self-transcendence trait: Toward an integrated framework through disaster psychology and a self-agency model

**DOI:** 10.3389/fnbeh.2022.943809

**Published:** 2022-08-18

**Authors:** Motoaki Sugiura

**Affiliations:** ^1^Institute of Development, Aging and Cancer, Tohoku University, Sendai, Japan; ^2^International Research Institute of Disaster Science, Tohoku University, Sendai, Japan

**Keywords:** adaptability, self-transcendence, spirituality, religiosity, self-agency, intentional binding, forward model, brain

## Introduction

Self-transcendence (ST) refers to expansion beyond the boundaries of the self in diverse dimensions, including physical and social. It often also includes expanded, prosocial, spiritual, and religious worldviews, as well as psychological and behavioral qualities that are achieved through ST (Garcia-Romeu, 2010). For empirical research of ST as a trait, many questionnaires have been developed (Kitson et al., 2020). In particular, the Self-Transcendence Scale (STS) (Reed, 1991) proposed in the field of nursing and the ST subscale of the Temperament and Character Inventory (TCI-ST) (Cloninger et al., 1993) in the field of psychobiology have contributed significantly to this research (Garcia-Romeu, 2010). These questionnaires conceptualize ST as the final stage of human psychological development and adaptability, particularly in old age.

However, the characteristics considered by these two ST trait questionnaires are dominated either by adaptability or supernaturalness. This may hinder the integration of this line of empirical research into the theoretical literature on ST, in which the coexistence of adaptability and supernaturalness is taken for granted (Yaden et al., 2017; Kaufman, 2020). The STS was developed to measure adaptive psychological and behavioral traits in older adults in the terminal stages of illness. Its items mainly evaluate connectedness and are intuitively acceptable to most people as adaptive (Reed, 1991). Only 1 (“finding meaning in my spiritual beliefs”) of 15 items has a slight supernatural nuance, which is inadequate for researchers who are interested in the relationship between the ST trait and spirituality or religiosity. Empirical studies that have used the STS have reported an association between scores and well-being in a variety of populations, including healthy young adults, and an increase in scores due to health-related vulnerability and age. Based on these findings, a model has been proposed in which ST moderates the negative impact of vulnerability on well-being (Reed, 2013). In contrast, the TCI-ST includes many items with supernatural, spiritual, and religious nuances, probably due to the multidimensional nature of TCI and the need for differentiation from other adaptive dimensions. Because of its uniqueness, the TCI-ST has gained significant attention and has been used in many studies. There is, however, little evidence of an association between TCI-ST scores and adaptability, such as well-being (Cloninger and Zohar, 2011; Spittlehouse et al., 2014; Moreira et al., 2015). Instead, many studies have reported an association between TCI-ST scores and psychotic traits (MacDonald and Holland, 2002; Ohi et al., 2012; Gaweda et al., 2015).

Although some cognitive bias is assumed to underlie the multiple dimensions of ST, its cognitive and neural bases are unknown. Previous research has focused mainly on the supernatural aspects of ST, considering them inseparable from spirituality and religiosity (MacDonald and Holland, 2002; Urgesi et al., 2010; Kitson et al., 2020). Anthropologically, these traits are considered to be linked to a higher-level cognitive bias inherent in humans (Bulbulia, 2004; Boyer and Bergstrom, 2008), such as the imagination that enables the formation of transcendent societies based on essentialized roles and groups (Bloch, 2008). Many neuroimaging studies have addressed the neural correlates of ST in terms of the experience or trait; however, an integrated view has yet to be achieved. Studies on spiritual or religious supernatural experiences are abundant, reporting diverse and different activation areas across studies (Rim et al., 2019; Kitson et al., 2020). Two studies have addressed the trait of ST or religiosity; notably, they found associations with decreased brain activity (Kapogiannis et al., 2009) and brain damage (Urgesi et al., 2010) of partially overlapping areas.

This paper uses recent findings in disaster psychology and the neurocognitive model of self-agency to consider whether adaptability and supernaturalness coexist in ST traits, as well as to evaluate the common cognitive bias and its neural basis that underlie the multifaceted nature of the ST trait.

## Do adaptability and supernaturalness coexist?

Recent disaster psychology research has identified ST trait concepts that include supernatural nuances. In a study that explored the psycho-behavioral characteristics that were advantageous for survival (Power to Live; P2L) among the survivors of the 2011 Great East Japan Earthquake (Sugiura et al., 2015a), eight factors were identified, including one that was consistent with ST, which consisted of the following four items (P2L-ST):

- I am aware that I am alive, have a sense of responsibility in living.
- I am aware of the path and teachings I should follow as a person.
- I am aware of the role I should play in society.
- I think that my actions towards others will go around and eventually come back to me.

It is noteworthy that items 1 and 4 have a supernatural nuance and overlap with the TCI-ST items. These items overlap with the representative items (i.e., with high loadings) of another ST questionnaire constructed in Japan from the viewpoint of transpersonal psychology (Nakamura, 1998).

**Figure 1:**
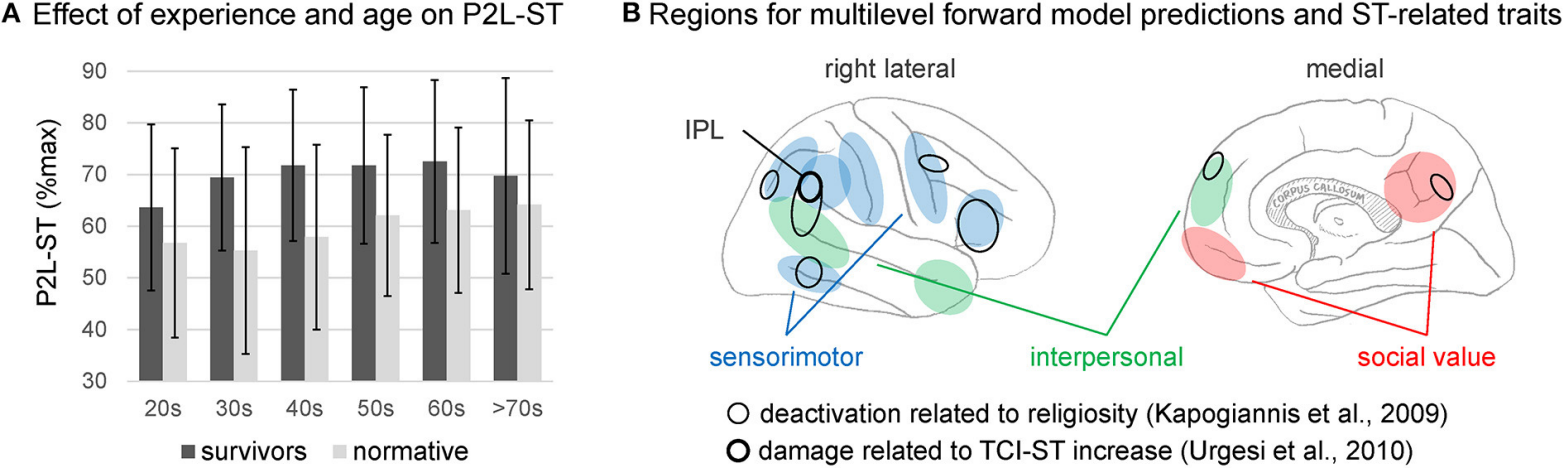
**(A)** Average and standard deviation (error bar) of the score (%max) of self-transcendence (ST) factor of the power to live (P2L) questionnaire separately for six age levels (from 20s to over 70) and two groups: disaster survivors (dark gray) and normative population (light gray). **(B)** Cortical regions implicated in forward model prediction in the three-layer model of self-agency (Sugiura13) and those implicated in ST-related traits. Regions are schematically drawn on the lateral (left panel) and medial (left panel) surface of the right cerebral hemisphere. Regions for the sensorimotor, interpersonal, and social value levels in the three-layer model of self-agency are given in blue, green, and red, respectively. Damage related to the increase in the ST score of the TCI [Temperament and Character Inventory Cloninger et al., 1993] (Urgesi et al., 2010) and deactivation related to a higher perceived level of God's involvement (Kapogiannis et al., 2009) are encircled by thick and thin lines, respectively.

The P2L-ST has also been demonstrated to have adaptability in terms of moderating the relationship between vulnerability and well-being, as proposed for STS in a nursing theory (Reed, 2013). The effect of vulnerability on P2L-ST seems evident, given the significant association of scores with disaster experience and age (Figure 1A) when the P2L-ST completed by members of the general population (*n* = 1200) (Ishibashi et al., 2019) was compared with data from disaster survivors (*n* = 1350) (Sugiura et al., 2015a). A three-way analysis of variance of disaster experience (two levels) × age (20s to >70s; six levels) × sex (two levels) showed medium (η^2^ > 0.06) and small (η^2^ > 0.01) main effect sizes for disaster experience [*F*_(1, 2526)_ = 190.629, *p* < 0.001, η^2^ = 0.068] and age [*F*_(5, 2526)_ = 10.562, *p* < 0.001, η^2^ = 0.019]; I inferenced significance using the effect size η^2^ (Cohen, 1992) rather than *p*-value, considering the large sample size. For the further details of the data, analysis, or results, see Supplementary Tables S1, S2. A relationship between P2L-ST and well-being has also been demonstrated. P2L-ST scores are associated with housing reconstruction and well-being during the reconstruction phase in survivors who have lost their housing; this association is not observed in survivors who have not lost their housing (Sato et al., 2021). Scores have also been reported to be positively correlated with helping behavior during tsunami evacuation (Sugiura et al., 2020).

## Cognitive and neural bases

Recent studies have suggested that the cognitive bias underlying the ST trait is related to the sense of self-agency based on forward model predictions. A study of the relationship between intentional binding, an established objective measure of the sense of self-agency in action, and the eight factors of the P2L identified a significant positive correlation between intentional binding and P2L-ST (Niikuni et al., 2022). Intentional binding is the process or degree of bias by which the time interval between one's action and the consequent sensory input is perceived to be shortened by forward model prediction; this is considered to be related to the sense of self-agency, particularly at the unconscious level (Haggard, 2017). Considering that intentional binding creates an, arguably illusory, consciousness of the relationship between the self and the external world, it may be a common cognitive basis of ST and a sense of self-agency. Intentional binding is also related to various adaptive traits. Strong intentional binding correlates with the belief in free will (Aarts and van den Bos, 2011), while weak intentional binding is associated with various negative psychological conditions, such as schizophrenia (Graham-Schmidt et al., 2016), obsessive-compulsive tendencies (Oren et al., 2019), and narcissism (Render and Jansen, 2019). Notably, similar to the TCI-ST, an association between intentional binding and psychotic tendencies (Graham et al., 2015) has been reported.

Does the cognitive bias, which is apparently limited to the sensorimotor domain, give rise to the multidimensionality of ST, including various social domains? Recent theories of social cognition and developmental psychology allow such conceptual expansion. It has been proposed that the forward model prediction process for the sense of self-agency in action allows for the development of the ability to perceive interactional relationships between the self and others (sense of shared agency) through repeated social interactions during infancy (Gergely, 2001). In line with this, studies have demonstrated a relationship between intentional binding and the sense of shared agency (Obhi and Hall, 2011), as well as between low intentional binding and low theory of mind ability in autism spectrum disorders (Zalla et al., 2015). Furthermore, a three-layer model of the sense of self-agency (Sugiura, 2013), inspired by the theory of adolescent developmental psychology (Cooley, 1902; Mead, 1934), suggests an extension of the forward model prediction process not only from the sensorimotor (action agency) to interpersonal (shared agency) levels but also to the social-value level, which concerns the awareness of one's social role and value in the larger social context, and may be related to the prosocial and moral dimensions of ST.

This conceptual expansion appears to be supported by neurobiological findings related to the ST trait, which imply a link between the ST trait and the mechanisms that inhibit multilevel forward model prediction and resulting error detection. In two previous studies on the trait of ST or religiosity, ST was associated with reduced brain activity or brain damage. Indeed, in general, a sense of self-agency is associated with reduced activity in brain regions involved in forward model prediction or related error detection. Damage to the inferior parietal lobule (IPL) is associated with elevated TCI-ST scores (Urgesi et al., 2010); this region has been implicated in prediction or error detection at the sensorimotor level (Schnell et al., 2007; Kikuchi et al., 2019). While thinking about religious beliefs, a relatively lower degree of activation has been identified in various cortical areas in individuals with a higher perceived level of God's involvement (Kapogiannis et al., 2009). The areas distributed over the lateral and medial cortex of the right cerebral hemisphere overlap with the regions for forward model prediction at the sensorimotor level (including the IPL), as well as at higher levels (i.e., interpersonal and social values) in the three-layer model of the sense of self-agency (Sugiura, 2013) (Figure 1B).

## Discussion and conclusion

The ST concept identified in recent disaster psychology research (P2L-ST) was thus found adaptive in terms of moderating the relationship between vulnerability and well-being, and included moderate supernatural nuances. The common cognitive bias underlying the multidimensionality of ST has been suggested to be related to a sense of self-agency, indicating the possibility that the bias is caused by a process that controls the neural networks involved in multilevel forward model prediction.

The latter conceptualization may allow for the understanding of individual differences in a variety of ST-relevant beliefs, such as cultural and religious beliefs, according to a recent theoretical framework of the believing process (Sugiura et al., 2015b). The framework attributes the characteristics of the believing process (e.g., self-organization and stability) to the structure of the belief representations composed of perceptual, action, and value components; the associations between the former two make up the very basis of the forward-model prediction. This framework also assumes a hierarchically nested structure of the representations in the three levels (Sugiura, 2013). Individual conformity to supernatural beliefs may be explained by the individual strength of common cognitive bias prevalent across multi-level believing processes.

These findings and hypotheses may also facilitate anthropological discussions of the development of human-specific sociality and culture, including religion, starting from the ST trait. Supernaturalness seems to be key to relating the ST concept to unique natures of religion and culture, and adaptability is the premise for discussing it in the context of evolution and development. Future discussions are expected as to whether anthropological hypotheses on the development of human-specific societies and cultures, including religion (Bulbulia, 2004; Bloch, 2008), are consistent with the notion of common cognitive bias between ST and the sense of self-agency, and with the neurocognitive hypothesis on the notion based on multilevel forward model prediction and its control process.

Several issues remain unaddressed. First, the relationship between the ST trait and psychosis requires further investigation. Although P2L-ST has not been examined regarding this issue, intentional binding, which correlates with the ST trait, is correlated with psychosis (Graham et al., 2015). The apparently contradictory associations may be because supernatural beliefs are adaptive only at a moderate level or because supernatural beliefs are an adaptive response to internal psychological or neurological adversity. Second, relationships between diverse supernatural, mystical, and religious experiences and beliefs, which are extensively evaluated in the TCI-ST, and the adaptability identified in the P2L-ST, are also uninvestigated. The implications of the neural activity reported in various brain regions in relation to supernatural experiences and beliefs also remain to be elucidated. Finally, the process through which the ST trait is enhanced by vulnerability is unknown. The process seems to be multiphasic; in the short term, intentional binding is weakened by negative events (Obhi et al., 2013) before the facilitatory effect of vulnerability on ST becomes apparent in the long term.

## Author contributions

MS conceptualized, analyzed the data, and wrote the paper.

## Funding

This study was supported by KAKENHI (20K20292) from the Japan Society for the Promotion of Science, and the Subsidy for Interdisciplinary Study and Research Concerning COVID-19 from the Mitsubishi Foundation. The publication of this paper is funded by Rüdiger Seitz, *via* the Volkswagen Foundation, Siemens Healthineers, and the Betz Foundation. The funders were not involved in the study design, collection, analysis, interpretation of data, the writing of this article or the decision to submit it for publication.

## Conflict of interest

The author declares that the research was conducted in the absence of any commercial or financial relationships that could be construed as a potential conflict of interest.

## Publisher's note

All claims expressed in this article are solely those of the authors and do not necessarily represent those of their affiliated organizations, or those of the publisher, the editors and the reviewers. Any product that may be evaluated in this article, or claim that may be made by its manufacturer, is not guaranteed or endorsed by the publisher.
