# Supplementary material for: Adaptability, supernaturalness, and the neurocognitive basis of the self-transcendence trait: Toward an integrated framework through disaster psychology and a self-agency model
Source: Front Behav Neurosci. 2022 Aug 18;16:943809. doi: 10.3389/fnbeh.2022.943809 (PMC9435587; doi:10.3389/fnbeh.2022.943809)
Supplement: Supplementary file 1 [file Data_Sheet_1.PDF]

Supplementary Material

Supplementary Table S1 Sample size of P2L-ST

group	sex	age					
		20s	30s	40s	50s	60s	>70s
survivors	male	39	57	76	106	145	123
	female	48	87	136	164	232	137
normative	male	100	100	100	100	100	100
	female	100	100	100	100	100	100

Sample size is given for each cell of the 2 (group) × 6 (age) × 2 (sex) three-way analysis of variance. The sample size of the disaster survivors was reduced from 1412 to 1350 due to the missing data necessary for the current analysis. See original papers (Ishibashi et al., 2019; Sugiura et al., 2015) for the details of the survey and profiles of the respondents (including the disaster-related damages in the survivors). The raw data is also available as supplementary materials of these papers.

Supplementary Table S2 Results of 3-way ANOVA on P2L-ST

		df	*F*	*p*	η^2	
main effect	group	1	190.629	<0.001	0.068	**
	age	5	10.562	<0.001	0.019	*
	sex	1	17.476	<0.001	0.006	
2-way interaction	group × age	5	3.972	0.001	0.007	
	group × sex	1	0.713	0.398	<0.001	
	age × sex	5	1.489	0.190	0.003	
3-way interaction	group × age × sex	5	1.705	0.130	0.003	
error		2526				
total		2549				

F-value, *p*-value, and η^2 are given for the main effects, two-way, and three-way interactions. Effect size η^2 (Cohen, 1992) rather than *p*-value was used for statistical inference considering the large sample size. **:$\eta^2 > 0.06$ (medium effect size), *:$\eta^2 > 0.01$ (small effect size).

References

Cohen, J. (1992). Quantitative methods in psychology: A power primer. *Psychological Bulletin*, *112*, 155–159.

Ishibashi, R., Nouchi, R., Honda, A., Abe, T., & Sugiura, M. (2019). A concise psychometric tool to measure personal characteristics for surviving natural disasters: Development of a 16-item Power to Live questionnaire. *Geosciences*, *9*(9), 366. https://doi.org/10.3390/geosciences9090366

Sugiura, M., Sato, S., Nouchi, R., Honda, A., Abe, T., Muramoto, T., & Imamura, F. (2015). Eight personal characteristics associated with the power to live with disasters as indicated by survivors of the 2011 Great East Japan earthquake disaster. *PLOS ONE*, *10*(7), e0130349. https://doi.org/10.1371/journal.pone.0130349
